# Comparison of Two Hepatitis B Vaccination Strategies Targeting Vertical Transmission: A 10-Year Japanese Multicenter Prospective Cohort Study

**DOI:** 10.3390/vaccines9010058

**Published:** 2021-01-17

**Authors:** Koji Nishimura, Keiji Yamana, Sachiyo Fukushima, Kazumichi Fujioka, Hiroshi Miyabayashi, Masao Murabayashi, Ken Masunaga, Aya Okahashi, Nobuhiko Nagano, Ichiro Morioka

**Affiliations:** 1 Department of Pediatrics and Child Health, Nihon University School of Medicine, Tokyo 1738610, Japan; nishimura.koji@nihon-u.ac.jp (K.N.); okahashi.aya@nihon-u.ac.jp (A.O.); nagano.nobuhiko@nihon-u.ac.jp (N.N.); 2 Department of Pediatrics, Kobe University Graduate School of Medicine, Kobe 6500017, Japan; k-yamana@kjf.biglobe.ne.jp (K.Y.); sachi4@med.kobe-u.ac.jp (S.F.); fujiokak@med.kobe-u.ac.jp (K.F.); 3 Department of Pediatrics, Kakogawa Central City Hospital, Kakogawa 6758611, Japan; 4 Department of Pediatrics, Kasukabe Medical Center, Kasukabe 3448588, Japan; miyabayashi@dr.memail.jp; 5 Department of Pediatrics, Numazu City Hospital, Numazu 4100302, Japan; numazuhp2451.shouni@bz04.plala.or.jp; 6 Division of Neonatology, Tokyo Metropolitan Ohtsuka Hospital, Tokyo 1708476, Japan; ken_masunaga@tmhp.jp

**Keywords:** hepatitis B surface antigen, hepatitis B virus, immunogenic response, mother-to-child infection, prevention

## Abstract

In 1985, a hepatitis B (HB) vaccination strategy against vertical HB virus transmission was introduced in Japan that recommended vaccination of infants at two, three, and five months of age (delayed strategy). This schedule was revised in 2013, recommending to vaccinate at birth and at 1 and 6 months of age (non-delayed strategy). We aimed to compare the vertical HB virus transmission rates and immunogenic responses between these two vaccination strategies. This Japanese multicenter prospective cohort study included 222 infants born between 2008 and 2017 to serum hepatitis B surface (HBs) antigen (HBsAg)-positive mothers. During the study period, 136 and 86 infants received delayed and non-delayed strategies, respectively. A positive vertical HB virus transmission was defined as a positive serum HBsAg status. Seropositive immunogenic response was defined as a serum anti-HBs titer of ≥10 mIU/mL. Post-vaccination serum HBsAg positivity rates did not differ significantly between the delayed (0/136 [0.0%, 95% confidence interval, 0.0–2.7%]) and non-delayed (2/86 [2.3%, 95% confidence interval, 0.3–8.1%]) strategy groups. Seropositive immunogenic response rates were 100.0% (136/136) and 97.7% (84/86), respectively. Although this study was under-powered to detect a statistically significant result, no vertical HB virus transmission was observed in the delayed strategy.

## 1. Introduction

Mother-to-child vertical transmission is a major route of hepatitis B (HB) virus infection. Therefore, several strategies to prevent vertical HB virus transmission are being implemented in many countries including Japan. However, in some countries excluding Japan, both the intramuscular HB immune globulin (HBIG) and the intramuscular HB vaccine are administered within 24 h after birth in infants born to serum HB surface antigen (HBsAg)-positive mothers. Thereafter, the second and third HB vaccine doses are administered at one to two months of age and at one year of age (non-delayed strategy), respectively [1,2]. This non-delayed strategy has been shown to prevent vertical HB virus transmission in 94–98% of infants born to serum HBsAg- and/or HB envelope antigen (HBeAg)-positive mothers [3,4,5,6] and to lead to 93–94% of seroprotective anti-HBs response rates after completion of the HB vaccination schedule [3,6].

**Figure 1:**
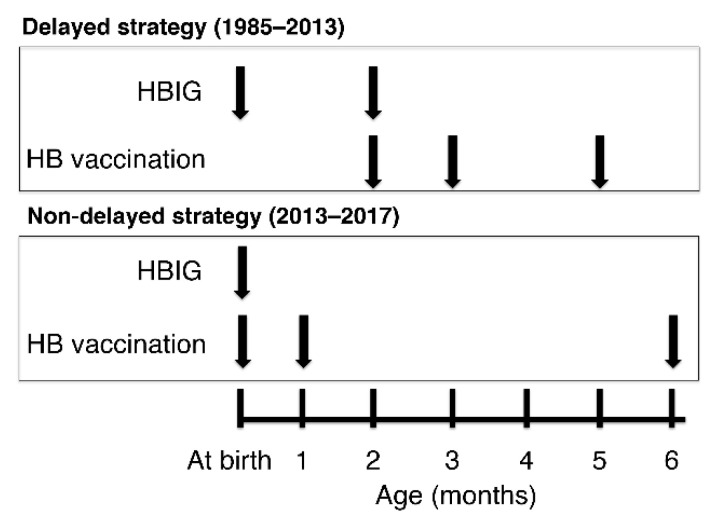
Delayed and non-delayed HB vaccination strategies against mother-to-child HB virus transmission in Japan. HB, hepatitis B; HBIG, hepatitis B immune globulin.

Since 1985, the delayed HB vaccination strategy, scheduled as three subcutaneous doses of HB vaccine at two, three, and five months of age with two intramuscular HBIG doses at birth and two months of age, was adopted to prevent vertical HB virus transmission (delayed strategy; Figure 1) among infants born to serum HBsAg- and HBeAg-positive mothers in Japan [7,8]. The schedule at birth was only HBIG without the HB vaccine, because the HB vaccine at that time was derived from plasma, and immunogenic responses were not as good as the current recombinant HB vaccine. Since 1995, this vaccination program has been made mandatory for all infants born to HBsAg-positive mothers in Japan, regardless of the mothers’ serum HBeAg status. In Japanese clinical studies, this delayed strategy has been shown to prevent mother-to-child HB virus transmission in more than 90% of infants [9,10] and to lead to 98–100% seroprotective anti-HBs response rates after the completion of the HB vaccination course [10,11].

The non-delayed strategy, with only one HBIG administration, is considered more economically rational and beneficial than the delayed method. Therefore, on 18 October 2013, the Ministry of Health, Labor, and Welfare in Japan approved the non-delayed strategy to prevent vertical HB virus transmission under the government health insurance coverage. Since its introduction, no studies have been conducted to evaluate this non-delayed strategy in Japanese clinical practice.

The aim of this Japanese multicenter prospective cohort study was to determine whether the non-delayed strategy—which is now widely used in Japan—is as effective in preventing mother-to-child vertical HB virus transmission and immunogenic responses as is the delayed strategy. In addition, we also investigated any difference in the effectiveness of genotype C- (serotype adr) and A-derived vaccines (serotype adw).

## 2. Materials and Methods

### 2.1. Study Design and Subjects

**Figure 2:**
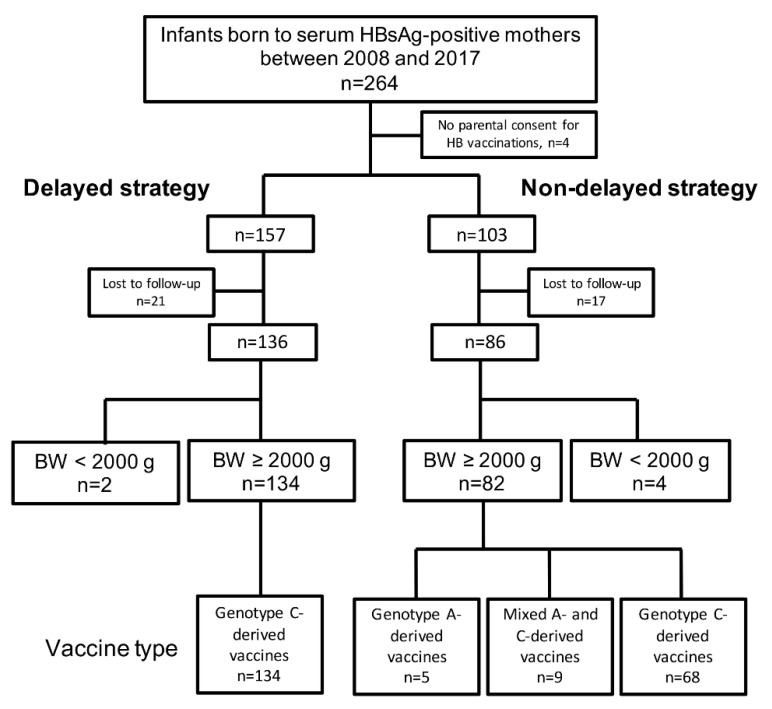
Flow of subjects. BW, birth weight; HB, hepatitis B; HBsAg, hepatitis B surface antigen.

This 10-year multicenter prospective cohort study included 264 infants born to serum HBsAg-positive mothers between 2008 and 2017 at six hospitals (Nihon University Itabashi Hospital, Kobe University Hospital, Kakogawa Central City Hospital, Kasukabe Medical Center, Numazu City Hospital, and Tokyo Metropolitan Ohtsuka Hospital) in Japan. Four infants of parents who refused to consent for HBIG and HB vaccinations and 38 infants who were lost to follow-up were excluded. Thus, 222 infants (136 and 86 infants received delayed and non-delayed HB vaccination strategies, respectively) were enrolled in this study (Figure 2).

### 2.2. Methods

#### 2.2.1. Delayed and Non-Delayed Strategies

For infants with birth weight ≥ 2000 g, delayed and non-delayed strategies were adopted in Japanese clinical practice from 1985 to 2013 and from 2013 to 2017, respectively (Figure 1). As per the delayed strategy, infants born to serum HBsAg-positive mothers received HBIG at birth (<48 h after birth) and at two months of age and thereafter received HB vaccines at two, three, and five months of age [7,8,9,10,11]. For the non-delayed strategy, infants received HBIG at birth (<12 h after birth) and HB vaccines at birth (<12 h after birth) and at one and six months of age [2,12]. HBIG was injected intramuscularly into the right and left femoral muscles (200 U/mL in total, consisting of 100 U/0.5 mL on each side). A genotype C- or A-derived HB vaccine (0.25 mL) was injected subcutaneously into the upper arm or femur (genotype C- derived vaccine: Bimmugen^®^, KM Biologics, Kumamoto, Japan and genotype A-derived vaccine: Heptavax^®^-II, MSD, Tokyo, Japan).

Infants with birth weight < 2000 g in the delayed strategy group received HBIG and HB vaccines as per the delayed strategy schedule [8]; however, for those in the non-delayed strategy group, HBIG and HB vaccines were administered as per the non-delayed strategy schedule with an additional dose at two months of age resulting in a four-dose (at birth [within 12 h after birth], 1-, 2-, and 6-months of age) vaccination course [2,13,14].

#### 2.2.2. Evaluation for Prophylaxis Success or Failure

The standard age at which serum HBsAg and anti-HBs titers were tested was six months for the delayed strategy group and 9–12 months for the non-delayed strategy group according to the Japan pediatric society guidance [12]. The prophylaxis was considered successful when the serum of an infant was found negative for HBsAg and the anti-HBs titer was ≥10 mIU/mL (a seropositive immunogenic response to vaccine) [5,8,12,15,16]. However, if the serum of an infant was positive for HBsAg, the prophylaxis was defined as a failure [6].

#### 2.2.3. Measurement Methods and Cut-off Values for Serum HB Markers

Serum HBsAg was measured using chemiluminescent enzyme immunoassay (CLEIA) or chemiluminescent immunoassay (CLIA) with commercially available kits (LumipulsePresto^®^ HBsAg-HQ, Fujirebio, Inc., Tokyo, Japan or Architect^®^ HBsAg QT Abbott, Abbott Japan Co., Ltd., Matsudo, Japan) according to the manufacturer’s instructions (the cut-off value was <0.05 IU/mL, respectively).

Serum anti-HBs titer was measured using CLEIA or CLIA with commercially available kits (LumipulsePresto^®^ HBsAb-N, Fujirebio, Inc., Tokyo, Japan or Architect^®^ HBs Antibody kit Orsab Abbott, Abbott Japan Co., Ltd., Matsudo, Japan) according to the manufacturer’s instructions (the cut-off value was 10 mIU/mL for a positive response; see Section 2.2.2, above).

Serum anti-HBc titer was measured using CLEIA with commercially available kits (LumipulsePresto^®^ HBcAb-III, Fujirebio, Inc., Tokyo, Japan) according to the manufacturer’s instructions (the cut-off value was <1.0 of the cut-off index).

Serum HBeAg was measured using CLEIA or CLIA with commercially available kits (Lumipulse^®^ I HBeAg-HQ, Fujirebio, Inc., Tokyo, Japan or Architect^®^ HBe Antigen Abbott, Abbott Japan Co., Ltd., Matsudo, Japan) according to the manufacturer’s instructions (the cut-off value was <1.0 of the cut-off index).

Serum anti-HBe titer was measured using CLEIA or CLIA with commercially available kits (Lumipulse^®^ HBeAb-N, Fujirebio, Inc., Tokyo, Japan or Architect^®^ HBe Antibody Abbott, Abbott Japan Co., Ltd., Matsudo, Japan) according to the manufacturer’s instructions (the cut-off value for the inhibition rate was <50%).

Serum HB virus DNA (HBV-DNA) was measured using quantitative real-time polymerase chain reaction with commercially available kits (Cobas TaqMan^®^ HBV “Auto” v2.0 or Cobas^®^ 6800/8800 System HBV, Roche Diagnostics K.K., Tokyo, Japan) according to the manufacturer’s instructions (the cut-off value [Log copy/mL] was “Not detected”).

#### 2.2.4. Study Variables and Main Outcome

Data of the following background characteristics were collected for the enrolled mothers (during pregnancy) and infants and compared between the delayed and non-delayed strategy groups: serum levels of HBsAg, anti-HBs titer, HBeAg, and HBV-DNA, gestational age at birth, birth weight, and sex of infants. The main outcomes, serum positive HBsAg and positive anti-HBs titer rates after completing the HB vaccines, were compared between the delayed and the non-delayed strategy groups. Infants with birth weight ≥ 2000 g and <2000 g received different doses of the HB vaccination; therefore, serum positive HBsAg and anti-HBs titer rates in each birth weight group were analyzed. In infants with birth weight ≥ 2000 g, the non-responder, low responder, medium responder, and high responder groups were defined as having negative HBsAg with anti-HBs titer < 10 mIU/mL, 10–299 mIU/mL, 300–999 mIU/mL, and ≥1000 mIU/mL, respectively. The percentages were compared between the delayed and non-delayed strategy groups. When classified as per the HB vaccine type (genotype C-derived, A-derived, or mixed), positive anti-HBs titer rates after completion of the HB vaccination course were analyzed. The mixed vaccine type was the mixed use of A- and C-derived vaccines.

### 2.3. Statistical Analyses

Data are shown as number (percentage) or median value (minimum to maximum value). For statistical analyses, Fisher’s exact test and Mann–Whitney U test were used to compare the two groups, and the χ^2^-test was used to compare multiple groups using the JMP^®^ Pro14 (SAS Institute Inc., Cary, NC, USA). A 95% confidence interval (CI) was analyzed by a Clopper–Pearson exact test. A *p* value < 0.05 was considered to be statistically significant.

## 3. Results

### 3.1. Background Characteristics in Mothers and Infants

The background characteristics of the mothers and infants are shown in Table 1. No significant differences were found in the background characteristics between the delayed and non-delayed strategy groups (Table 1). No significant differences (*p* = 0.48) emerged in the number of infants lost to follow-up between the delayed (21/157, 13.4%) and non-delayed strategy groups (17/103, 16.5%). Furthermore, no significant differences were found in the background characteristics of the mothers and infants lost to follow-up between the delayed and non-delayed strategy groups (Table 2).

### 3.2. Serum Positive HBsAg and Positive Anti-HBs Titer Rates after Completing the HB Vaccines

The standard evaluation age for prophylaxis was predetermined in the Japan pediatric society guidance [12] to be six (5–13) months and 11 (8–21) months for the delayed and non-delayed strategy groups, resulting in a significantly earlier evaluation in the former than in the latter (*p* < 0.0001). In infants who completed the HB vaccination schedule, the positive HBsAg rates were 0.0% and 2.3% and the positive anti-HBs titer rates were 100.0% and 97.7% for the delayed and non-delayed strategy groups, respectively (Table 3). In infants with birth weight ≥2000 g, no significant differences were observed in the positive HBsAg and anti-HBs titer rates between the two strategy groups. Prophylaxis was successful for the six infants with birth weight < 2000 g (Table 3). The HB virus-related marker results in two infants with the positive HBsAg (prophylaxis failure) are shown in Table 4.

### 3.3. Serum Anti-HBs Titer Levels in Infants with Birth Weight ≥ 2000 g

After excluding the six infants with birth weight < 2000 g due to the small sample size, no significant differences were found in the percentages of non-responders, low responders, medium responders, and high responders between the delayed and non-delayed strategy groups in the 216 infants with birth weight ≥ 2000 g (Table 5).

### 3.4. Positive Anti-HBs Titer Rates after Completing the Genotype C- or A-Derived HB Vaccines

The infants with birth weight ≥ 2000 g (n = 216), were further analyzed to examine the differences between groups receiving genotype C- or A-derived HB vaccines. In the delayed strategy group, all (100.0%) 134 infants were vaccinated with C-derived vaccine. However, in the non-delayed strategy group, 68 (82.9%) received C-derived vaccine, 9 (11.0%) received a mixed dose of A- or C-derived vaccines, and 5 (6.1%) received A-derived vaccine (Figure 2). Two infants with prophylaxis failure received a mixed dose of A- or C-derived vaccines in the non-delayed strategy group.

Both delayed and non-delayed strategies with exclusively C-derived vaccine led to 100.0% positive anti-HBs titer rates (134/134 and 68/68, respectively).

In the non-delayed strategy group, positive anti-HBs titer rates were 68/68 (100.0%, 95% CI, 94.7–100.0%) in the C-derived vaccine group, 7/9 (77.8%, 95% CI, 40.0–97.2%) in the mixed A- and C-derived vaccine group, and 5/5 (100.0%, 95% CI, 47.8–100.0%) in the A-derived vaccine group.

## 4. Discussion

The age at which the HB virus is first transmitted to a person is a determinant of subsequent chronic HB infections (HB virus carrier). When HB virus is transmitted during the perinatal period or up to one year of age, more than 90% of the infants become HB virus carriers [2]. In the present study, the prophylaxis success rate was 100% for the delayed strategy group and 98% for the non-delayed strategy group; indicating that a high prophylaxis success rate can be obtained even when the HB vaccination is administered at birth and only one dose of HBIG is administered instead of two.

We also confirmed seropositive immunogenic responses in infants with birth weights of <2000 g, whose antibody-producing ability is considered to be immature [17]; two of these infants received the vaccinations as per the delayed strategy, whereas four received them as per the non-delayed strategy. This corroborates the findings of our recent report, wherein a seropositive immunogenic response rate of 96% was observed among 55 infants with birth weight < 2000 g who received vaccinations as per the delayed strategy [8].

Prophylaxis failure generally occurs in approximately 5% of infants who receive vertical HB transmission preventive measures [4,5]. In the present study, two infants developed prophylaxis failure in the non-delayed strategy group (prophylaxis failure rate: 1% in all subjects and 2% in the non-delayed strategy group). A possible explanation could be an intrauterine HB transmission or prophylaxis failure despite the vertical HB transmission preventive measures.

Intrauterine HB infection occurs at a frequency of 2–5% [2]. A high serum HBV-DNA level in the HBeAg-positive mother is a risk factor for intrauterine infection; the current vertical HB transmission preventive measures are incapable of preventing in utero transmission [5]. Recently, the use of tenofovir, a nucleotide analog, to pregnant women with positive HBeAg and high HBV-DNA load during pregnancy has been reported to have a preventive effect on intrauterine HB infection [18]. The World Health Organization has recently recommended that HBsAg positive pregnant women with a high HBV-DNA load receive tenofovir prophylaxis from the 28th week of pregnancy until at least birth [19], although it has not been recommended in current Japanese guidelines yet [20]. Apart from intrauterine HB infection, the host and virus factors are two other causes of prophylaxis failure in infants despite reception of vertical HB transmission preventive measures. Regarding the host factor, Nishida et al. reported that the DRB1-DQB1 haplotypes on the human leukocyte antigen class II region are specifically involved in refractory cases of the HB vaccine [21]. Regarding the virus factor, there exists a strain referred to as the HB vaccine-escape mutant that has an amino acid in the region responsible for HBV neutralizing antibody production, called “a” epitope of the major S protein, mutated from glycine to arginine [22,23]. For the two infants with prophylaxis failure in our study, we could not determine if the HB infection was caused in utero or prophylaxis failure occurred despite the vertical HB transmission preventive measures. Unfortunately, the mothers’ and infants’ genes and the viruses were not closely examined.

Due to the prevalence of HB virus with genotype C in Japan [24], the genotype C-derived vaccine is widely used in the country. As the rate of acute hepatitis due to genotype A HB virus, which is generally prevalent in Europe and the United States, has been recently increasing in Japan [25], the genotype A-derived vaccine is also in circulation since 2014. The mixed use of A- and C-derived vaccines began with the introduction of the A-derived HB vaccine in Japan in the non-delayed strategy era. A small Japanese clinical study by Komatsu et al. confirmed that anti-HBs titers were greater than 100 mIU/mL in 100% (7/7) of the infants who received the mixed use of A- and C-derived vaccines [26]. Tregnaghi et al., reported that the anti-HBs titer levels from different HBV genotype-derived vaccines (one dose of genotype A-derived vaccine and two doses of genotype C-derived vaccines) are equivalent to those of the same genotype-derived vaccinations in the non-delayed strategy group [27]. Contrastingly, two out of nine infants in our study who received the mixed A- and C-derived vaccines in the non-delayed strategy group had prophylaxis failure.

There are several limitations to the present study. First, because the entry criterion was only serum positive for HBsAg in the mother, not all enrolled mothers were examined for all the HB virus-related markers. Second, infants receiving genotype A-derived vaccine were few; because genotype C-derived vaccines are more commonly used in Japanese clinical practice. Third, since the HB virus markers were measured at each facility, the measurement method could not be unified. However, HB virus markers, especially anti-HBs titer levels, have been reported to have very good correlations between the measurement methods based on the kits used in Japan, and the errors are small [28]. Fourth, the evaluation age of anti-HBs titer levels was different between the delayed and non-delayed strategy groups. Ideally, the evaluation age should be the same, but this limitation could not be overcome because standard evaluation ages are predetermined by the Japanese clinical guidance. Fifth, because there were only two infants with the prophylaxis failure in our present study, we did not statistically compare the vertical HB virus transmission rates between these two vaccination strategies. Finally, any infection in the delayed group was not observed. The reasons might be that genotype C-derived vaccine, which is more adapted to the Japanese context, was used for all infants, the timing of evaluation was earlier, and less sensitive HBsAg measurement kits were used in the delayed group, because this was an observational study using a historical comparison group.

## 5. Conclusions

In this 10-year Japanese multicenter prospective cohort study, the seropositive immunogenic responses of vertical HB virus transmission prevention measures were comparable for infants receiving vaccinations as per the delayed and non-delayed strategies. Although this study was under-powered to detect statistically a significant result, no vertical HB virus transmission was observed in the delayed strategy.

## Figures and Tables

**Table 1:** Background characteristics of the mothers and infants, who were enrolled in this study.

Variables	All Subjects n = 222	Delayed Strategy n = 136	Non-Delayed Strategy n = 86	*p* Value
Mother				
Positive HBsAg rate	222/222 (100.0%)	136/136 (100.0%)	86/86(100.0%)	
HBsAg levels, IU/mL				
0.05–1999	38/142 (26.7%)	23/84 (27.3%)	15/58 (25.8%)	0.99
≥2000	104/142 (73.2%)	61/84 (72.6%)	43/58 (74.1%)	
Positive anti-HBs titer rate	5/155 (3.2%)	3/88 (3.4%)	2/67 (3.0%)	0.88
Anti-HBs titer level, mIU/mL	0.3 (0.0–250)	0.3 (0.0–250)	0.4 (0.0–20)	0.83
<10	48/52 (92.3%)	32/34 (94.1%)	16/18 (88.9%)	0.90
10–299	4/52 (87.7%)	2/34 (5.9%)	2/18 (11.1%)	
300–999	0 (0.0%)	0 (0.0%)	0 (0.0%)	
≥1000	0 (0.0%)	0 (0.0%)	0 (0.0%)	
Positive HBeAg rate	61/205 (29.8%)	41/126 (32.5%)	20/79 (25.3%)	0.30
HBeAg level, cut-off index	0.4 (0.2–2.297)	0.4 (0.2–1.885)	0.4 (0.2–2.297)	0.20
Positive anti-HBe titer rate	110/165 (66.7%)	60/98 (61.2%)	50/67 (74.6%)	0.87
HBV-DNA level, Log copy/mL	5.0 (2.1–9.0)	5.1 (2.1–9.0)	4.2 (2.1–9.0)	0.34
<2.1	10/87 (11.5%)	4/51 (7.8%)	6/36 (16.7%)	0.45
2.1–8.9	62/87 (71.3%)	36/51 (70.1%)	26/36 (72.2%)	
≥9.0	15/87 (17.2%)	11/51 (21.6%)	4/36 (11.1%)	
Infants				
Gestational age at birth, weeks	39 (25–41)	38 (27–41)	39 (25–41)	0.81
Birth weight, g	3077(918–4160)	3077(1212–4138)	3077(918–4160)	0.98
Male	117/222 (52.7%)	71/136 (52.2%)	46/86 (53.5%)	0.98

Data are shown as applicable number/available number (percent) or median value (minimum to maximum value). HBe, hepatitis B envelope; HBeAg, hepatitis B envelope antigen; HBs, hepatitis B surface; HBsAg, hepatitis B surface antigen; HBV-DNA, hepatitis B virus DNA.

**Table 2:** Background characteristics of the mothers and infants, who were lost to follow-up.

Variables	Subjects n = 38	Delayed Strategy n = 21	Non-Delayed Strategy n = 17	*p* Value
Mother				
Positive HBsAg rate	38/38 (100.0%)	21/21 (100.0%)	17/17(100.0%)	
HBsAg levels, IU/mL				
0.05–1999	6/35 (17.1%)	4/19 (21.1%)	2/16 (12.5%)	0.67
≥2000	29/35 (82.9%)	15/19 (78.9%)	14/16 (87.5%)	
Positive anti-HBs titer rate	0/14 (0%)	0/8 (3%)	0/6 (3%)	
Anti-HBs titer level, mIU/mL	0.35 (0.1–6.6)	1.3 (0.1–6.6)	0.25 (0.1–1.3)	0.15
<10	14/14 (100.0%)	8/8 (100.0%)	6/6 (100.0%)	
10–299	0 (0.0%)	0 (0.0%)	0 (0.0%)	
300–999	0 (0.0%)	0 (0.0%)	0 (0.0%)	
≥1000	0 (0.0%)	0 (0.0%)	0 (0.0%)	
Positive HBeAg rate	10/33 (30.3%)	5/19 (26.3%)	5/14 (35.7%)	0.71
HBeAg level, cut-off index	0.4 (0.2–1,681)	0.4 (0.2–1,443)	0.3 (0.3–1,681)	0.71
Positive anti-HBe titer rate	18/27 (66.7%)	10/15 (66.7%)	8/12 (66.7%)	1.00
HBV-DNA level, Log copy/mL	4.3 (2.1–9.0)	4.3 (2.6–7.6)	6.2 (2.1–9.0)	0.60
<2.1	0 (0.0%)	0 (0.0%)	0 (0.0%)	
2.1–8.9	12/15 (80.0%)	7/7 (100.0%)	5/8 (62.5%)	
≥9.0	3/15 (20.0%)	0 (0.0%)	3/8 (37.5%)	
Infants				
Gestational age at birth, weeks	39 (36–41)	40 (36–41)	39 (37–40)	0.13
Birth weight, g	3140 (2360–4025)	3146 (2775–4025)	3140 (2360–3755)	0.13
Male	18/38 (47.4%)	11/21 (52.4%)	7/17 (41.2%)	0.53

Data are shown as applicable number/available number (percent) or median value (minimum to maximum value). HBe, hepatitis B envelope; HBeAg, hepatitis B envelope antigen; HBs, hepatitis B surface; HBsAg, hepatitis B surface antigen; HBV-DNA, hepatitis B virus DNA.

**Table 3:** Serum positive HBsAg and positive anti-HBs titer rates after completion of the HB vaccination schedule.

Subjects		Delayed Strategy	Non-Delayed Strategy
All infants	N	136	86
	Positive HBsAg rate	0 (0.0%, 0.0–2.7%)	2 (2.3%, 0.3–8.1%)
	Positive anti-HBs titer rate	136 (100.0%, 97.3–100.0%)	84 (97.7%, 91.9–99.7%)
Infants with BW ≥2000 g	N	134	82
	Positive HBsAg rate	0 (0.0%, 0.0–2.7%)	2 (2.4%, 0.3–8.5%)
	Positive anti-HBs titer rate	134 (100.0%, 97.3–100.0%)	80 (97.6%, 91.5–99.7%)
Infants with BW <2000 g	N	2	4
	Positive HBsAg rate	0 (0.0%, 0.0–84.2%)	0 (0.0%, 0.0–60.2%)
	Positive anti-HBs titer rate	2 (100.0%, 15.8–100.0%)	4 (100.0%, 39.8–100.0%)

Data are shown as number (percent, 95% confidence interval). HBsAg, hepatitis B surface antigen; HBs, hepatitis B surface; BW, birth weight; N, number.

**Table 4:** The HB virus-related marker results in two infants with the positive HBsAg (prophylaxis failure).

Variables	Case 1	Case 2
Gestational age at birth	39 weeks	39 weeks
Birth weight	2716 g	3262 g
Gender	Male	Female
Year	2016	2016
Evaluation age	10 months	11 months
HBsAg levels, IU/mL	31,497	56,465
Anti-HBs titer level, mIU/mL	0.4	5.5
Anti-HBc titer	Positive	Positive
HBeAg level, cut-off index	1740	1490
Anti-HBe titer, the cut-off value for the inhibition rate	<1	<1
HBV-DNA level, Log copy/mL	Not examined	8.9
Genotype	Not examined	B

HB, hepatitis B; HBc, hepatitis B core; HBe, hepatitis B envelope; HBeAg, hepatitis B envelope antigen; HBsAg, hepatitis B surface antigen; HBs, hepatitis B surface; HBV-DNA, hepatitis B virus DNA.

**Table 5:** Serum anti-HBs titer levels in infants with birth weight ≥ 2000 g.

Anti-HBs Titer Level, mIU/mL	All Subjects n = 216	Delayed Strategy n = 134	Non-Delayed Strategy n = 82	*p* Value
<10	2 (0.9%)	0 (0.0%)	2 (2.4%)	0.45
10–299	56 (25.9%)	38 (28.4%)	18 (22.0%)	
300–999	92 (42.6%)	59 (44.0%)	33 (40.2%)	
≥1000	66 (30.6%)	37 (27.6%)	29 (35.4%)	

Data are shown as number (percent). HBs, hepatitis B surface.

## Data Availability

The data presented in this study are available in this article.
